# Characterization of *Escherichia coli* isolates associated with bovine mastitis in Austria

**DOI:** 10.3389/fvets.2026.1812989

**Published:** 2026-06-16

**Authors:** Susanna M. Piechl, Adriana Cabal Rosel, Werner Ruppitsch, Elke Müller, Sascha D. Braun, Stefan Monecke, Ralf Ehricht, Axel Wehrend, Verena Urbantke, Thomas Wittek, Michael P. Szostak, Martina Baumgartner, Igor Loncaric

**Affiliations:** 1 Clinical Department of Farm Animals and Food Systems Safety Clinical Center for Ruminant and Camelid Medicine University of Veterinary Medicine, Vienna, Austria; 2 Austrian Agency for Health and Food Safety (AGES), Institute for Epidemiology and Infectious Disease Surveillance, Vienna, Austria; 3 Institute of Hygiene and Medical Microbiology, Medical University Innsbruck, Innsbruck, Austria; 4 Faculty of Food Technology, Food Safety and Ecology, University of Donja Gorica, Podgorica, Montenegro; 5 Leibniz-Institute of Photonic Technology (Leibniz-IPHT), Member of the Leibniz Center for Photonics in Infection Research (LPI), Jena, Germany; 6 InfectoGnostics Research Campus Jena e.V., Jena, Germany; 7 Institute of Physical Chemistry, Friedrich-Schiller-University, Jena, Germany; 8 Faculty of Veterinary Medicine, Veterinary Clinic for Reproductive Medicine and Neonatology, Justus-Liebig-University Giessen, Gießen, Germany; 9 Department for Biological Sciences and Pathobiology, Institute of Microbiology, University of Veterinary Medicine, Vienna, Austria

**Keywords:** bovine mastitis, *E. coli*, MAEC, microarray-based diagnostics, resistance genes, virulence genes, whole genome sequencing (WGS)

## Abstract

Despite advances in dairy husbandry, mastitis-associated *Escherichia (E*.) *coli* (MAEC) remains a primary causative agent of bovine mastitis, contributing significantly to economic losses in the cattle industry. This study characterized 50 *E. coli* isolates from severe, moderate, mild, and subclinical mastitis by antimicrobial susceptibility testing and genotypic analysis, including PCR, DNA microarray, *E. coli* phylotyping and whole-genome sequencing of selected strains. Antimicrobial resistance rates were highest for ampicillin (30%), tetracycline (20%), amoxicillin-clavulanate (20%), and trimethoprim-sulfamethoxazole (14%); The majority of isolates (66%) was susceptible to antibiotics. One isolate displayed an ESBL phenotype, carrying *bla*_CTX-M-3_ and *bla*_TEM-1_ genes. Phylogenetic analysis indicated that members of phylogroups B1 (50%) and A (16%) are predominant among the isolates, followed by group C (12%), with the remaining isolates belonging to groups D, E, and F, and two unassigned. The most prevalent virulence-associated genes detected were *fimH* (100%), and genes of the ferric dicitrate uptake locus (*fecIRABCDE*). Overall, MAEC isolates exhibited diverse and heterogeneous genetic profiles. The proliferation of ESBL-producing *E. coli* poses a threat within a One Health framework, given the risk of multidrug-resistant strains at the human-animal-environment interface, compromising critical antimicrobials in both veterinary and human medicine.

## 1 Introduction

Mastitis is a leading cause of economic loss and health problems in dairy cattle and is a major reason for early culling (1). Antimicrobial treatment is often required to maintain udder health, animal welfare, and economic stability. However, the use of antimicrobials in food-producing livestock is a critically discussed issue due to the increasing development of antimicrobial resistance (2, 3).

*Escherichia* (*E*.) *coli* is an important and prevalent cause of environmental mastitis (4), which can cause intramammary infections ranging from severe but temporary inflammation to subclinical or mild clinical infections that are more chronic (1). *E. coli* strains are categorized as intestinal pathogenic (InPEC) or extraintestinal pathogenic (ExPEC), depending on their ability to colonize different anatomic sites (5, 6). *E. coli* isolates from bovine mastitis belong to the ExPEC group, which are part of the intestinal microflora where they colonize asymptomatically the gut. However, if they reach niches outside, they can act as pathogens. Some ExPEC are considered to share characteristic virulence factors and are therefore grouped into pathotypes (7, 8). In recent studies, molecular characterization of bovine mastitis-associated *E. coli* strains did not identify any specific virulence factors clearly associated with bovine mastitis (6, 8). The main virulence factors that have been identified so far in mastitis-associated *E. coli* strains include genes encoding adhesins/fimbriae (*fimH, fimA, sfaDE, papC, afaBcIII, iha*), invasins (*ibe*), toxins/hemolysins (*hlyA, vat, cnf, cdt*), and mechanisms for serum resistance (*kpsM*), which participates in the biosynthesis of group 2 and group 3 capsules, and siderophores (*iroN, iucD, iutA*) (9, 10).

The common feature of MAEC is rather an enrichment in fitness capabilities for survival and rapid adaptation in the mammary gland, which are referred to as intramammary ecotypes and is considered as mastitis-associated *E. coli* (6). The *E. coli* strains have a great intraspecific diversity and are classified into eight phylogenetic groups (A, B1, B2, C, D, E, F, and clade) (11, 12). The majority of mastitis-associated *E. coli* strains are clustered in group A and B1 (6). Regarding antimicrobial resistance, prevalence in mastitis strains is strongly influenced by strain origin and therefore varies considerably (6). Antimicrobial resistance is a significant threat to global food security, human health, and the future of livestock production. Overall, evidence indicates that antimicrobial resistance in mastitis-associated *E. coli* is a reactive process in response to antibiotic use (13). Thus, monitoring the resistance situation and the use of antibiotic agents in the dairy industry are essential. Changes in resistance patterns can occur unpredictably and pose a challenge for the entire ecosystem (14). Although previous studies have identified virulence-associated genes in MAEC, there is a notable research gap concerning these genes in Austria. The present study aims to characterize a collection of MAEC isolates from various locations within Austria. This study presents the first characterization of Austrian MAEC isolates and employs a One Health approach to address the interconnected risks these pathogens pose to animal welfare, economic stability, and public health.

## 2 Materials and methods

### 2.1 *E. coli* isolates

Fifty *E. coli* isolates were recovered from subclinical and clinical mastitis cases in dairy cows, using aseptically collected quarter milk samples. A mastitis case was defined as subclinical if milk was macroscopically normal, accompanied by an elevated somatic cell count. Clinical mastitis cases were defined as mild (Severity Score 1), if only milk secretions were abnormal. Cases with alterations of the milk and local inflammatory reactions (e.g., swelling of the affected quarter) defined a moderate (Score 2); anamnestic reports of systemic inflammatory reactions (e.g., fever) were defined as severe (Score 3) clinical mastitis, and subclinical mastitis (Score 4) (15). Isolates were identified as presumptive *E. coli* based on colony characteristics, and species identification was performed with MALDI-TOF mass spectrometry (Bruker Daltonik, Heidelberg, Germany). Isolates were stored at −80 °C for further analyses.

### 2.2 Antimicrobial susceptibility testing

Antimicrobial susceptibility was determined by the agar disk diffusion method (16), using the following agents: ampicillin (10 μg), amoxicillin-clavulanate (20/10 μg), cefotaxime (30 μg), ceftazidime (30 μg), cefoxitin (30 μg), meropenem (10 μg), gentamicin (10 μg), tobramycin (10 μg), amikacin (30 μg), ciprofloxacin (5 μg), trimethoprim-sulfamethoxazole (1.25/23.75 μg), tetracycline (30 μg), chloramphenicol (30 μg), fosfomycin (200 μg), and nitrofurantoin (300 μg) (all from Becton Dickinson, Heidelberg, Germany). *Escherichia coli* ATCC® 25922™ was used as a quality control strain. Extended-spectrum beta-lactamase (ESBL) production was assessed using combination disk tests with cefotaxime and ceftazidime (with or without clavulanic acid; Becton, Dickinson, Heidelberg, Germany). AmpC beta-lactamase production was screened using cefoxitin (30 μg).

### 2.3 Molecular characterization

All isolates underwent characterized as follows. *E. coli* phylogroups were determined as previously described (11, 12). A set of virulence-associated genes (Table 1) was determined using a custom-made DNA microarray-based technology from INTER-ARRAY (INTER-ARRAY by fzmb GmbH, Bad Langensalza, Germany) (17, 18). In addition, the following genes were screened via PCRs: *sfaDE, ihA,* and *afaBC III* (19, 20). The genes *fecI* and *fecE* of *fec* locus (*fecIRABCDE*), usually present in MAEC, were screened using primers designed during the present study (*fec*I primer: fecI-fwd: 5´-ATCACGGCTGGTTGAAAAG-3′ and fecI-rev: 5´-CAATCTCGCTGTATGTCAG-3′ (381-bp amplicon), and *fecE* primer: fecE-fwd: 5´-TGCCTCAGCACCATTTAAC-3′ and fecE-rev: 5´-CATTACCACCAGTTGATCG-3′ (392-bp amplicon)). Twenty *E. coli* isolates were selected for whole-genome sequencing according to clinical presentation (mostly severe clinical mastitis with fever, sometimes accompanied by gastrointestinal disorders) and resistance profile. Genomic DNA isolation, whole-genome sequencing, and subsequent genome assembly was conducted as described previously (21). Strain typing and serogenotype prediction were done in SeqSphere+ (Ridom, Münster, Germany), while phylogroups were assigned with the Clermont Typing tool (http://clermontyping.iame-research.center/, accessed on 2025 November 30) (22). Sequence types (STs) were identified in EnteroBase (23). SeqSphere + software (Ridom, Münster, Germany) was used for core-genome multilocus sequence typing (cgMLST) analysis. Resistance/virulence genes were identified using ABRicate (24) with ResFinder (25), Comprehensive Antibiotic Resistance Database CARD (26), and Virulence Factor Database (27). Additional gene prediction and annotation were performed using the Bacterial and Viral Bioinformatics Resource Center (BV-BRC) (28).

**Table 1:** A set of virulence-associated genes tested using DNA-microarray.

Virulence factor	Gene	Function
paa	*acfC*	Porcine attaching-effacing associated protein Paa/adherence factor adfo
aidA	*aidA*	Responsible for bacterial autoaggregation
bfpB	*bfpB*	Bundle forming pilus B
escV	*escV*	Type III secrection inner membrane protein
ent	*espL*	Ent protein
fimH	*fimH*	Typ I fimbrial protein
F5	*fimK99/fanC*	Fimbrial protein
exhA	*hlyA-var1*	Haemolysin
hlyA	*hlyA-*var*2*	Haemolysin
iucD	*iucD*	Aerobactin biosynthesis protein
papC	*papC*	Outer membrane usher P fimbriae
invE	*virB*	Enteroinvasive protein
aggR	*virF-aggR*	Regulator of enteroaggregative *E. Coli*
astA	*astA*	Heat-resistant agglutinin, EAST
bfpA	*bfpA*	Bundle forming pilus A
cnf1	*cnf*	Cytotoxic necrotizing factor
eaeA	*eae*	Intimin for attaching and effacing
F17	*f17aG*	Fimbrial protein for adhesion
F6	*fasA*	Fimbrial protein for adhesion
F18	*fedA*	Fimbrial protein for adhesion
F41	*fim-41a*	Fimbrial protein for adhesion
F4	*fim-K88ab/faeG*	Fimbrial protein for adhesion
gad	*gad*	Transcriptional regulator, important in acid environment
elt, LT (eltA)	*ltcA-var1*	Heat-labile enterotoxin A subunit
pic	*pic*	Serine protease of enteroaggregative *E. Coli*
estIa	*sta1*	Heat-stabile enterotoxin ST-Ia
estIb	*sta2*	Heat-stabile enterotoxin II
stx1	*stx1*	Shigatoxin
stx2	*stx2*	Shigatoxin
Stx2e	*stx2e*	Shigatoxin

## 3 Results

### 3.1 Mastitis score

All 50 isolates were confirmed as *E. coli* by MALDI TOF MS. The majority of isolates (*n* = 39) originated from severe mastitis cases (Score 3), while the remaining eleven originated from subclinical (*n* = 3), mild (Score 1, *n* = 3), or moderate (Score 2, *n* = 5).

### 3.2 Antimicrobial susceptibility testing

All 50 *E. coli* isolates were susceptible to carbapenems, fosfomycin, and amikacin. Seventeen (34%) displayed resistance to at least one antimicrobial, and seven (14%) were classified as multidrug-resistant (29). No isolate showed AmpC beta-lactamase production, and one isolate (P22) exhibited an ESBL phenotype. Overall, 33 of 50 isolates (66%) were fully susceptible to all tested antimicrobials. Antimicrobial resistance rates were highest for ampicillin (30%), tetracycline (20%), amoxicillin-clavulanate (20%), and trimethoprim-sulfamethoxazole (14%). Tables 2 and 3 summarize these results.

**Table 2:** Phenotypic and genotypic characterization of *E. coli* isolates from bovine mastitis using whole-genome sequencing.

Sample number	Mastitis score	Phylogroup	Sequence type	Serotype	ResistancePhenotyp^1^	Resistance genotype	Virulence genes	Mutations QRDR^2^ GyrA	Mutations QRDR	Mutations QRDR	Biocide resistance genes
									ParC	ParE	
P1	4	B1	2,598	O175: H16			*fim* Operon,				
							*fecIRABCDE*				
P3	3	B1	1,125	O_NT_: H19	NR		*fim* Operon *astA, hlyA-var2, fecIRABCDE*				
P5	3	C	410	O_NT_: H9	AMP, AMC, FQR, TET, CHL, SXT	*bla*_OXA-1_*, aadA, aadA5, tet*(A)*, floR, sul1, sul2, dfrA17, dfrA36*	*fim* Operon, *astA,. papC1, papC2, incD1, iucD2, fecIRABCDE, sitABCD*	*gyrA* D87N, *gyrA* S83L	*parC*S80I	*parE*	*qacE*
										S458A	

P7	3	B1	18,185	O_NT_: H21	NR		*fim* Operon, *cnf1, f17, papC1, papC2, hlyA-var2, incD1, iucD2, iha, cdt, iutA, fecIRABCDE*				
P10	3	D	69	O15: H18	AMP, TET	*bla*_TEM-1_,	*fim* Operon, *fecIRABCDE sitABCD*				
						*tet*(A)					
P12	4	B1	1,396	O_NT_: H7	NR		*fim* Operon, *fecI, fecIRAB*				
P14	3	F	117	O_NT_: H4	AMP, AMC, TET	*bla*_TEM-1_, *aph*(3″)-Ib, *aph*(6)-Id, *tet*(A)*, sul2*	*fim* Operon, *astA, pic, papC1, papC2, iucD1, iucD2, vat, iroN, fecIRABCDE,* *sitABCD*	*gyrA* S83L			*D*
P17	3	A	744	O101: H9	AMP, AMC, FQR, TET, CHL, SXT	*bla*_OXA-1_*, aph*(3′)-Ia,	*fim* Operon, *papC1, papC2, iucD2, iha, fecIRABCDE,* *sitABCD*	*gyrA* D87N*, gyrA* S83L	*parC*A56T*, parC*S80I		*D, qacE*
						*aadA, aph*(3″)-Ib*, aph*(6)-Id, *tet*(B), *catA1, floR, sul1,*					
						*dfrA36*					

P20	1	E	1,508	O_NT_: H45	NR		*fim* Operon*, fecIRABCDE*				
P22	3	C	88	O8: H25	ESBL, GEN, TET, SXT	*bla*_CTX-M-3_, *bla*_TEM-1_*, aac*(3)-IId, *aph*(3′)-Ia, *aadA1, aadA5, tet*(B),	*fim* Operon, *astA, papC1, papC2 afaA-VIII/afaA-VII, afaD-VIII, afaE-VIII, fecIRABCDE, sitABCD*				*, qacE*
						*sul1, dfrA17*					

P27	3	B2	547	O_NT_: H5	NR		*fim* Operon*, vat, iroN, ibe, kpsM, kpsFEDUCS, fecI, fecE,*				
P28	3	A	10	O_NT_: H16	NR		*fim* Operon, *astA, cnf1, papC1, papC2, hlyA-var2, iucD1, iucD2, iha, cdt, afaA-VIII/afaA-VII, afaD-VIII, afaE-VIII, iutA, fecIRABCDE*				
P31	3	B1	58	O_NT_: H25	AMP, AMC, GEN, TOB, TET, CHL, SXT	*bla*_TEM-1_*, ant*(2″)-Ia	*fim* Operon, *cnf1, papC1, papC2, hlyA-*var*2, iucD1, iucD2, iroN, fecIRABCDE,* *sitABCD*				*, qacE*
						*ant*(2″)-Ia, *aadA1, aph*(3″)-Ib*, aph*(6)-Id					
						*ant*(2″)-Ia, *tet*(A),					
						*floR, sul1, sul2, dfrA5*					
P34	1	B2	73	O_NT_: H1	NR		*fim* Operon, *cnf1, pic, hlyA-var2, sfaDE, vat, iroN, kpsM, kpsFEDUCS, sfa, set, foc*, *fecIRABCDE,* *sitABCD*				
P35	2	B1	3,234	O_NT_: H8	AMP		*astA, fim* Operon, *K88ab (F4), fecIRABCDE*				
P37	3	B1	446	O88: H8	NR		*fim* Operon, *f17, fecIRA*				
P40	3	B1	2,521	O_NT_: H7	NR		*fim* Operon, *acfC, fecIRA*				
P42	3	B1	58	O_NT_: H16	NR		*fim* Operon, *f17, afaD-VIII, afaE-VIII, fecIRABCDE*				
P44	3	A	18,186	O1: H21	NR		*fim* Operon, *aidA, fecIRABCDE*				
P49	1	B1	10	O_NT_: H32	NR		*fim* Operon*, fecIRABCDE,* *sitABCD*				

^1^AMC, amoxicillin and clavulanate; CAZ, ceftazidime; CHL, chloramphenicol; CIP, ciprofloxacin; CFZ, cefazolin; CTX, cefotaxime; FOF, fosfomycin; GEN, gentamicin; PIP, piperacillin; SXT, trimethoprim & sulfamethoxazole; TET, tetracycline; TOB, tobramycin; NR, not resistant; ^2^QRDR: quinolone-resistanc-determining region.

**Table 3:** Phenotypic and genotypic characterization of *E. coli* isolates from bovine mastitis using Microarray technology and PCRs.

Sample number	Mastitis score	Phylotype	Resistance phenotyp^1^	Virulence genes
P2	3	NT	NR	*astA, fimH1, fimH2, hlyA-var2, fecI, fecE*
P4	3	D	AMP, TET	*fimH1, fimH2, iha, cnf1, hlyA-var2, fecI, fecE*
P6	3	B1	NR	*fimH1, fimH2, hlyA-var2, fecI, fecE*
P8	3	A	NR	*fimH1, fimH2, astA, fecI, fecE*
P9	3	B1	NR	*fimH1, fimH2, fecI*
P11	3	E	NR	*fimH1, fimH2, fecI, fecE*
P13	3	C	AMP, AMC, FQR, TET, CHL, SXT	*fimH1, fimH2, astA, iha, papC1, papC2, iucD1, iucD2, fecI, fecE*
P15	3	C	AMP, AMC, FQR, TET, CHL, SXT	*fimH1, fimH2, fecI, fecE*
P16	3	A	NR	*fimH1, fimH2, fecI, fecE*
P18	3	B1	NR	*fimH1, fecI, fecE*
P19	3	B1	NR	*fimH1, fecI, fecE*
P21	3	A	NR	*fimH1, fecI, fecE*
P23	3	B1	AMP	*fimH1, fecI*
P24	3	B2	NR	*fimH1, fecI, fecE*
P25	3	B1	AMP	*fimH1, fimH2, astA, hlyA-var2, fecI, fecE*
P26	3	C	NR	*fimH1, fimH2, fecI, fecE*
P29	3	C	AMP, AMC, FQR, TET, CHL, SXT	*fimH1, fimH2, astA, iha, papC1, papC2, iucD1, iucD2, fecI, fecE*
P30	3	B1	NR	*fimH1, fimH2, fecI*
P32	2	B1	NR	*fimH1, fimH2, astA, fecI, fecE*
P33	3	B1	NR	*fimH1, fimH2, f17, fecI, fecE*
P36	2	B1	AMP, AMC	*fimH1, fimH2, astA, hlyA-var2, fecI, fecE*
P38	2	A	NR	*fimH1, fimH2, fecI, fecE*
P39	2	B1	NR	*fimH1, fimH2, fecI, fecE*
P41	3	B1	NR	*fimH1, fimH2, fecI*
P43	3	B1	NR	*fimH1, fimH2, cnf1, papC1, papC2, hlyA-var2, iucD1, iucD2, fecI, fecE*
P45	3	B2	NR	*fimH1, fimH2, cnf1, hlyA -var2, fecE*
P46	4	B1	FOS	*fimH1, fimH2, sfaDE, fecI, fecE*
P47	3	B1	AMP, AMC, TET	*fimH1, fimH2, cnf1, f17, papC1, papC2, hlyA-var2, iucD1, iucD2, fecI, fecE*
P48	3	NT	NR	*fimH1, fecE*
P50	3	A	NR	*fimH1, fimH2, fecI, fecE*

^1^AMP, ampicillin; AMC, amoxicillin-clavulanate; CAZ, ceftazidime; CHL, chloramphenicol; CIP, ciprofloxacin; CFZ, cefazolin; CTX, cefotaxime; FOF, fosfomycin; GEN, gentamicin; PIP, piperacillin; SXT, trimethoprim-sulfamethoxazole; TET, tetracycline; TOB, tobramycin; NR, not resistant.

### 3.3 Molecular characterization of *E. coli*

The predominant phylogenetic group was B1 (*n* = 25, 50%), followed by A (*n* = 8, 16%), C (*n* = 6, 12%), and B2 (*n* = 4, 8%). Groups D and E each comprised two isolates (4% per group), and group F was represented by only one isolate (2%). Two isolates could not be assigned to any known phylogroup.

Twenty selected isolates underwent WGS, revealing 16 distinct sequence types (STs). ST10 and ST58 were each detected in two isolates (10% each), while the remaining STs (ST2598, ST1125, ST410, ST69, ST1396, ST117, ST744, ST1508, ST88, ST547, ST73, ST3234, ST446, ST2521, and two new ST18186 and ST18185) were singletons. No clustering was observed by cgMLST. WGS-based serotype analysis identified six distinctive O: H serogenotypes. Thirteen strains were O-non-typeable, and 12 distinct H-types were observed. Two multidrug-resistant isolates (P22, P31) belong to serotype O8: H25. Isolate P22 (phylogroup C, ST88) was ESBL-producing (*bla*_CTX-M-3_ and *bla*_TEM-1_) and resistant to gentamicin, tetracycline, and trimethoprim-sulfamethoxazole, while P31 (phylogroup B1, ST58) contained *bla*_TEM-1_ was resistant to was resistant to ampicillin, amoxycillin-clavulanic acid, gentamicin, tobramycin, tetracycline, chloramphenicol, and trimethoprim-sulfamethoxazole. P5 (ONT: H9, C, ST410) carried *bla*_OXA-1_ and mutations in quinolone-resistance-determining region (QRDR) (*gyrA* D87N, *gyrA* S83L, *parC* S80I, *parE* S458A). P17 (O101: H9, A, ST744) had *bla*_OXA-1_ with QRDR mutations (*gyrA* D87N, *gyrA* S83L, *parC* A56T, *parC* S80I). All four isolates were multidrug-resistant. P10 (phylogroup D, ST69) with the serotype O15: H18 carried the *bla*_TEM-1_ gene. P14 (ONT: H4, phylogroup F, ST117) carried *bl*a_TEM-1_ and *gyrA* S83L. Phenotypic resistance, which is well reflected by the observation that the isolates carried various AMR genes: tetracycline resistance genes (*tet*(A), *tet*(B), trimethoprim-sulfamethoxazole resistance genes (*sul1, sul2, dfrA17, dfrA36, dfrA5*), chloramphenicol resistance genes (*floR, catA1*) and aminoglycoside resistance gene (*aac (3)-IId, ant(2″)-Ia, aph(3′)-Ia, aadA1, aadA5, aph(3″)-Ib, aph (6)-Id*). Biocide resistance genes were found in 4/20 isolates: four carried *qacE. I*solates carried distinct virulence-associated genes. The *fec* operon was detected in 15 of 20 (75%) isolates in this study. Whole-genome sequencing revealed that all strains harbor *fecIRABCDE*, except for four strains. Strains P37 and P40 carry *fecIRA*, followed by a plasmid insertion, resulting in the absence of additional *fec* genes. Isolate P12 possesses *fecIRAB*, with a subsequent deletion extending to one epimerase gene. Strain P27 lacks all *fec* genes (Table 2). Among isolates that were whole-genome sequenced, the most prevalent virulence-associated genes were those in the *fim* operon (95%, 19/20). Less frequent genes included *iroN* (20%), *vat* (15%), *cdt* (10%), *ibeA* (5%), and *kpsM* (10%). The adhesins *afaD-VIII* and *afaE-VIII* were observed in 3/20 (15%) isolates. One isolate showed the fimbrial adhesin genes *foc* and *sfa*. Finally, eight isolates carried *sitABCD* (iron uptake/transport relevant). The genes for the identification of enteroaggregative *E. coli astA* and *pic* were also detected using a DNA microarray (Table 1). Using a DNA microarray, all isolates carried the *fimH1* gene; 41 (82%) harbored *fimH2*. The virulence gene *papC* was present in 11 isolates (22%), while *iucD* (siderophore biosynthesis) was detected in 10 isolates (20%). Hemolysin gene *hlyA* was detected in 20 (40%), and *f17* in five (10%). Each of the VAGs *acfC, aidA*, and K88 was detected in a single isolate. *fecI* (48/50, 96%) and/or *fecE* (43/50, 86%)—genes associated with ferric dicitrate uptake (Tables 2, 3).

## 4 Discussion

The aim of the present study was to characterize phenotypically and genetically mastitis-associated *E. coli* isolates from clinical and subclinical mastitis cases in Austria using a polyphasic approach that included susceptibility testing, PCR, DNA microarray-based technology, and WGS. The *E. coli* isolates examined showed genetic heterogeneity, reflected in the distribution of phylogroups and distinct STs, as well as in the variable presence of virulence genes. The virulence genes detected occurred in different combinations, showing a flexible set of pathogenicity factors.

Most isolates originated from severe clinical mastitis cases. The majority of isolates were assigned to phylogroups B1 and A, and lower rates were assigned to phylogroups C, B2, E, and F, consistent with previous studies (6, 9, 30).

The MAEC isolates analyzed in the present study exhibited sequence type (ST) diversity, reflecting substantial genetic variability within the selected population. Due to differences in the molecular methods applied to date (PCR, MLST, DNA microarray, whole-genome sequencing), it is often challenging to compare distinct MAEC virulence and resistance genes and their STs. ST10, ST58, ST88, ST117 and ST1125 found in our study, were also identified in isolates from different farms and with varying scores of mastitis (10, 30),and all these STs have multiple entries (bovine/mastitis) in the Enterobase *Escherichia/Shigella* Database (https://enterobase.warwick.ac.uk/species/index/ecoli, last accessed 18. November 2025). The affiliations of ST10, ST58, ST410, and ST73 are often associated with pandemic high-risk ExPEC strains that can survive in various ecological niches, including the human gastrointestinal tract and the environments of domestic and farm animals (31). One of the most characteristic genes we detected, which is observed frequently in MAEC and UPEC genomes than in other *E. coli* strains, is the fec operon (*fecIRABCDE*), which encodes the ferric dicitrate transport system (10, 32, 33)and represents an important molecular determinant contributing to the pathogenicity of MAEC. Isolate P7, with a new sequence type ST18185, carried previously detected genes in MAEC (*papC, hlyA, cnf1, cdt, iucD, iutA*), *fecIRABCDE*, and adhesin gene f17, which transmits adhesion to host cells. f17 are frequently found in ETEC strains and in up to 20% of MAEC isolates, depending on the study (6). The adhesin gene *fimH* was consistently detected across all isolates. Beyond this gene, different STs harbored distinct arrays of VGs associated with previously reported ExPEC pathotypes (UPEC and MAEC) (6, 9). ExPEC-associated VGs such as *hlyA* (haemolysin), *papC* (P-fimbriae), *iucD* (siderophore synthesis), and *cnf1* (cytotoxic necrosis factor 1) were present in 16 to 24% of isolates, as well as *astA*, a virulence-associated gene associated with enteroaggregative *E. coli*. Other VAGs, including adhesins (*sfaDE, iha*) and toxin or siderophore genes (*vat, cdt, iroN*), were less common, consistent with previous MAEC studies (6, 9, 10).

Specifically, 33 isolates (66%) were phenotypically susceptible to all tested antibiotics, while seven of the fifty isolates (14%) exhibited a multidrug-resistant (MDR) profile. The highest resistance rates were observed for ampicillin (30%), tetracycline (20%), amoxicillin-clavulanate (20%), and trimethoprim-sulfamethoxazole (14%), consistent with data reported for MAEC isolates from Belgium (ampicillin 28.8%), France (amoxycillin/clavulanic acid 22%; tetracycline 20%), and Portugal (trimethoprim-sulfamethoxazole 13.1%) (34).

Genotypic analysis of *β*-lactamase genes revealed *bla*_TEM-1_ in three isolates (ST58, ST69, ST117), *bla*_CTX-M_-3/*bla*_TEM-1_ in one multidrug-resistant isolate (ST88), and *bla*_OXA-1_ in two isolates (ST410, ST744). The prevalence of β-lactamase and ESBL genes (*bla*_CTX-M_, *bla*_TEM_, and occasionally *bla*_OXA_) in MAEC varies considerably across geographical regions (34, 35). The multidrug-resistant isolates also showed phenotypic and genotypic resistance to tetracyclines (*tet*(A), *tet*(B)) and trimethoprim-sulfamethoxazole (*sul1, sul2, dfrA17, dfrA36, dfrA5*), commonly observed in *E. coli* from farm animals due to the widespread veterinary use of these antibiotics (34, 36).

The strains P5 (ST410), P17 (ST744), P22 (ST88) and P31 (ST58) harbored genotypically the biocide resistance genes *qacE* (Quaternary Ammonium Compound Efflux Gene) encode efflux pumps that confer tolerance to quaternary ammonium compounds (QACs), and the antibiotic resistance genes *sul1* and *aadA* which are often found together with on class 1 integrons, promoting co-selection (37). Eight isolates carried the operon *sitABCD*, originally described as a metal ion transporter that contributes to survival in iron-poor environments such as the mammary gland and improves fitness in ExPEC isolates during infection by enhancing iron uptake, as with siderophore systems such as *iucD* or *iroN* (38). The co-occurrence with *qacE* in mastitis-associated strains could indicate adaptation to multiple traits, such as environmental persistence (*qacE*) or fitness for colonization of the host (*sitABCD*) (38). The limitation of this study is the lack of phenotypic testing for biocide resistance patterns.

The presence of high-risk pandemic sequence types and multidrug-resistant strains in Austrian MAEC highlights a One Health concern regarding pathogen transmission between livestock, the environment, and humans. Mitigating these strains is both a public health priority and an economic necessity, essential for reducing production losses and ensuring the dairy sector’s financial sustainability.

The number and regional distribution of tested strains were insufficient to determine the nationwide prevalence of resistance and virulence genes. Due to variation in virulence factors assessed worldwide and the high heterogeneity of these strains, it is challenging to establish uniform characteristics for this pathotype. Furthermore, investigation of the entire *fec* operon was limited by the availability of primers only for *fecI* and *fecE*. An additional limitation of this study is the lack of phenotypic testing for biocide resistance patterns. Future studies should incorporate a broader range of isolates from all Austrian localities and provide more comprehensive characterization, such as analyzing all isolates using whole genome sequencing rather than a selected subset.

## 5 Conclusion

This study is the first in Austria to conduct a genotypic analysis of mastitis-associated *E. coli* strains. The aim was to provide an overview of potential virulence mechanisms and regional antimicrobial resistance patterns associated with bovine mastitis, a severe disease affecting dairy cows. The isolates showed a high genetic variability. The occurrence of typical ExPEC VAGs such as *fimH, fimA, papC, hlyA, cnf1, vat, iroN*, and *iucD*, as well as the *fec* operon, underscores the importance of characterization of bovine *E. coli* isolates originating from mastitis. The detection of ESBL-producing isolates in milk represents a significant public health concern, reinforcing the need for integrated One Health surveillance programs. The identification of the fec operon and specific multidrug-resistant sequence types provides actionable targets for the development of regional diagnostic tools and the refinement of targeted antimicrobial therapies to reduce economic losses in Austrian dairy farming.

## Data Availability

The original contributions presented in the study are publicly available. This data can be found here: NCBI Sequence Read Archive, accession PRJNA1471175.
